# Supplementary material for: OpenMM 7: Rapid development of high performance algorithms for molecular dynamics
Source: PLoS Comput Biol. 2017 Jul 26;13(7):e1005659. doi: 10.1371/journal.pcbi.1005659 (PMC5549999; doi:10.1371/journal.pcbi.1005659)
Supplement: S1 Source Code — (ZIP) [file pcbi.1005659.s002.zip › OpenMM-7.0.1-Source/docs/api-c++/generated/OpenMM.Integrator.html]

Integrator — OpenMM @OPENMM\_MAJOR\_VERSION@.@OPENMM\_MINOR\_VERSION@ documentation


# Integrator¶

*class* `OpenMM::``Integrator`¶
:   An `Integrator` defines a method for simulating a `System` by integrating the equations of motion. This is an abstract class. Subclasses define particular integration methods.

    Each `Integrator` object is bound to a particular `Context` which it integrates. This connection is specified by passing the `Integrator` as an argument to the constructor of the `Context`.

    Methods

    |  |  |
    | --- | --- |
    | `Integrator` |  |
    | `~Integrator` |  |
    | `getStepSize` | Get the size of each time step, in picoseconds. |
    | `setStepSize` | Set the size of each time step, in picoseconds. |
    | `getConstraintTolerance` | Get the distance tolerance within which constraints are maintained, as a fraction of the constrained distance. |
    | `setConstraintTolerance` | Set the distance tolerance within which constraints are maintained, as a fraction of the constrained distance. |
    | `step` | Advance a simulation through time by taking a series of time steps. |

    `Integrator`()¶

    `~Integrator`()¶

    double `getStepSize`() *const*¶
    :   Get the size of each time step, in picoseconds. If this integrator uses variable time steps, the size of the most recent step is returned.

        |  |  |
        | --- | --- |
        | Returns: | the step size, measured in ps |

    void `setStepSize`(double *size*)¶
    :   Set the size of each time step, in picoseconds. If this integrator uses variable time steps, the effect of calling this method is undefined, and it may simply be ignored.

        |  |  |
        | --- | --- |
        | Parameters: |  |

        - **size** – the step size, measured in ps

    double `getConstraintTolerance`() *const*¶
    :   Get the distance tolerance within which constraints are maintained, as a fraction of the constrained distance.

    void `setConstraintTolerance`(double *tol*)¶
    :   Set the distance tolerance within which constraints are maintained, as a fraction of the constrained distance.

    void `step`(int *steps*) = 0¶
    :   Advance a simulation through time by taking a series of time steps.

        |  |  |
        | --- | --- |
        | Parameters: |  |

        - **steps** – the number of time steps to take

High performance molecular simulation on GPUs


### Navigation

- Core Objects
- Forces
- Integrators
- Extras

©2015, Stanford University and the Authors.
|
Powered by Sphinx 1.3.1
& Alabaster 0.7.7
|
Page source
